# A cohort-based assessment of drug use trends during the COVID-19 pandemic: relationship with mood and sociodemographic factors in Brazil

**DOI:** 10.3389/fpsyt.2025.1514365

**Published:** 2025-01-30

**Authors:** Nubia Heidrich, Maurício Schüler Nin, Felipe Borges Almeida, Hilda M. R. M. Constant, Luana Freese, Helena M. T. Barros

**Affiliations:** ^1^ Graduate Program in Health Sciences, Universidade Federal de Ciências da Saúde de Porto Alegre, Porto Alegre, Brazil; ^2^ Neuropsychopharmacology Laboratory, Department of Pharmacosciences, Universidade Federal de Ciências da Saúde de Porto Alegre, Porto Alegre, Brazil; ^3^ Department of Behavioral Science Methodology, Universitat de València, València, Spain

**Keywords:** substance-related disorders, alcohol, tobacco, illicit drugs, longitudinal study, depression, anxiety, COVID-19 pandemic

## Abstract

The COVID-19 pandemic has brought significant challenges, including severe psychological consequences, especially for vulnerable individuals, such as those with substance use disorders. This study investigated the impact of the pandemic on substance use patterns and psychological health in Brazilians, exploring associations with sociodemographic factors to identify groups at higher risk. Data were collected online to assess self-reported substance use through the Alcohol, Smoking and Substance Involvement Screening Test (ASSIST), and psychological state, using the Depression, Anxiety and Stress Scale (DASS-21) and level of social distancing. The research was conducted in three waves: September-October 2020, April-May 2021, and September-November 2022. The ASSIST (alcohol, cannabis, hallucinogens, and cocaine/crack) and DASS-21 (anxiety, depression, and stress) scores decreased over time. Regarding sociodemographic data, being male, single, with less education, lower income and lower social distancing showed associations with alcohol and cannabis scores. All drug scores showed associations with psychological symptoms and time, suggesting a possible adaptation or resilience of the sample to the challenges of the pandemic. These findings highlight the importance of monitoring patterns of substance use and mental health in times of crisis, especially in vulnerable populations. Such knowledge is essential to inform public health strategies and prepare health systems to face future global crises.

## 1 Introduction

The Coronavirus Disease 2019 (COVID-19) pandemic is an ongoing global health concern, with 746.41 million clinical cases and 6.93 million deaths worldwide being recorded between its beginning in late December of 2019 and towards the late stage of the epidemic in May of 2023 (1, 2). During this time, social distancing measures have been implemented by the government of several countries with the aim of slowing the spread of the infectious agent and to release the overburden on health systems. In Brazil, a nationwide quarantine law was implemented on March 20, 2020 and lasted for several months (3). After the many hardships related to social isolation, fears of a deadly disease, uncertainties regarding the disease itself, need to help others and doubts as to the efficacy of the different vaccines, by the end of the pandemic there were around 13.6 billion vaccinated individuals worldwide (1).

During this period, news outlets began reporting on how the lockdown and social isolation could affect the mental health of the population (4, 5). Special concern was raised for individuals presenting vulnerabilities such as mental disorders, neurological or physical diseases, or living in a harmful, unsafe or distressing socioeconomic environment. Several studies tried to unveil the relationship between the many factors related to the pandemic and mental health (6–9). It was observed that loneliness, psychological distress and weak social support networks brought direct negative consequences to certain groups. For instance, COVID-19 infected patients, students, financially vulnerable individuals, elderly and pregnant, caretakers, and, including people who misuse drugs and people living with addiction (10–14). At that particular time, questions regarding increase or decrease in use and abuse of several legal and illegal drugs were of notable interest due to concerns of worse outcomes—including death—when this group was infected with COVID-19 (15–17). In fact, a meta-analysis demonstrated increased risk for hospitalization and increased mortality risk after COVID-19 infection in people with any mental disorder and substance use disorder (11).

In this context, the mental health symptoms that are most frequently studied are stress, anxiety and depression, and to lesser extents, sleep disturbances, post-traumatic stress disorder, eating disorders, among others. Given the timely need for research on the impact of the COVID-19 pandemic on drug abuse and dependence, a previous study from our research group reported cross-sectional data from the Brazilian population with the aim of investigating a potential link between social distancing levels and drug use patterns. We found that social distancing level was inversely associated with increased self-perception of drug use (13) and that emotional state presented associations with the Alcohol, Smoking and Substance Involvement Screening Test (ASSIST, version 3.1) scores (18) for almost all drugs. Furthermore, gender, income and education showed associations with a variety of drug classes scores as well (13). Concerning longitudinal studies that reach to 2022, although not a large body of literature, we found evidence suggesting the amelioration of psychological outcomes that were worse in the beginning of the pandemic in different populations (19, 20). Considering the relevance of these findings, the follow up of the individuals evaluated during the pandemic published in Nin et al. (13), is necessary to understand the influence between these two factors: psychological post-pandemic adaptation and drug use changes after that period. This study aimed to investigate the impact of the pandemic on substance use patterns and psychological health of Brazilians.

## 2 Methods

### 2.1 Design and participants

This study is a cohort with participants recruited in a cross-sectional survey conducting annual data collections during the pandemic. The first cohort time point (C1) occurred between September 9th and October 16th, 2020. Data for the second time point (C2) was collected between April 29th and May 22th, 2021. The third and final cohort time point (C3) was conducted between September 9th and November 21th, 2022, six months after the revocation of the Brazilian National Public Health Emergency, following the increase in vaccination. For further recruitment details, see the original cross-sectional study (13). We chose these specific timepoints taking local government-issued measures and the rise in vaccinated people into consideration. To be eligible, all participants had to be ≥ 18 years old and residing in Brazil, and each questionnaire had to be completed to the last question. The study was submitted in the National Ethics Committee Platform (“Plataforma Brasil” registry number: 5.376.167) and approved by the Ethics Committee of Research of the Federal University of Health Sciences of Porto Alegre (#4241378).

### 2.2 Instruments and scales

All follow-ups were collected using the Research Electronic Data Capture (REDCap^®^, Vanderbilt University, Nashville, TN, USA) online platform. Participants who explicitly agreed to continue their participation for the next phases of the study were recruited to the subsequent stages through an individualized link that was sent to the e-mail address provided on C1. E-mail reminders were sent periodically according to overall response rate. The first page of the survey contained the informed consent with an accept/decline button, followed by the questionnaire on the next pages. It was composed of 56 questions and was divided into four sections (fully disclosed in **Supplementary Material**): 1) sociodemographic data; 2) social distancing and vaccination during the pandemic (questions originally created by authors); 3) the Brazilian-validated version of the ASSIST instrument (18, 21), chosen for its reliability and validity in measuring the use risks of psychoactive substances; 4) the Depression, Anxiety and Stress Scale (DASS-21), also validated in Portuguese (22). At the end of the survey, a counseling message based on the ASSIST calculated score was presented, with their risk of dependence being classified as low, moderate, or high. For moderate and high risk, participants were recommended to seek help from a health care practitioner.

### 2.3 Measures

Sociodemographic data were collected in categories, while age was a continuous variable. As determined in the first study published about these data, some sociodemographic strata were collapsed when subgroup samples were too small to be analyzed on their own. Education was collected into eight categories and collapsed into three, “Incomplete Secondary Education”, “Complete Secondary Education” and “Higher Education”. Income was collected in the local currency (Brazilian Real) (USD$ 1.00 ≅ R$ 5.00), and the minimum wage in Brazil during the cohort period ranged from R$ 1,045.00 to R$ 1,212.00. Social distancing perception regarded the level of restriction in social distancing assessed as three categories (Low, Medium and High). All sociodemographic factors were collected in the first survey filled by the subjects.

The Brazilian version of the ASSIST microstructured questionnaire included self-reported frequency of drug use, lifetime use, and use in the three months before the first pandemic wave (C1-September 9th to October 16th, 2020), the second wave (C2-April 29th and May 22nd of 2021), and around the end of the pandemic (C3-September 9th and November 21st of 2022) based on DSM-IV criteria for drug dependence. The score obtained for each substance falls into a “lower” (“occasional use”), “moderate” (“in risk for abuse”), or “high” risk (“dependence”) category which determines the most appropriate intervention for each categorical level of use. Scores from 0 to 3 are considered occasional use (for alcohol: 0 to 10); ≥ 4 to 26 indicates moderate risk for dependence (for alcohol: 11 to 26); ≥ 27 suggests dependence or high risk of dependence for alcohol and all other drugs (18).

The DASS-21 contemplated three subscales (depression, anxiety, and stress) with a Likert format, varying from 0 (did not apply to me at all) to 3 (applied to me very much or most of the time). Cut-offs for levels of subscales are presented as “Normal” - Depression: 0 to 9; Anxiety: 0 to 7; Stress: 0 to 14. “Mild” - Depression: 10 to 13; Anxiety: 8 to 9; Stress: 15 to 18. “Moderate” - Depression: 14 to 20; Anxiety: 10 to 14; Stress: 19 to 25; Severe- Depression: 21 to 27; Anxiety: 15 to 19; Stress: 26 to 33; “Extremely severe” - Depression: >28; Anxiety: >20; Stress: >34 (22).

### 2.4 Statistical analysis

The inferential analysis investigated the effect of time and sociodemographic factors in the ASSIST scores and DASS-21 scores, as well as the interaction between time and factors through Generalized Linear Models (GLM) or Two Way RM-ANOVA. *Post-hoc* comparisons were tested using the Bonferroni test or Tukey, respectively. To carry out the GLM, the scores for drug use (ASSIST) were considered the dependent variable. The independent variables were the sample profile (sociodemographic data) and the severity of social distancing. For identifying risks within the social distancing categories, the reference category was “very rigorous”. The covariates defined as controls in the models were listed based on the significant results observed in the bivariate analysis (comparisons of social distancing level with sociodemographic variables and DASS scale).

For GLM analysis, it was considered all the subjects who filled at least one of the surveys (C1 and C2 and/or C3) since this statistical approach allows the use of datasets with missing data, summing n = 2498. For the other statistical approaches, such as ANOVA, Chi-Square and Pearson Correlation, it was considered only the n = 755 who filled all surveys (C1, C2 and C3). Normality and equal variance tests were performed to verify if non-parametric tests were necessary.

The Chi-Square test was performed for dichotomized variables (DASS-21 severity rating prevalence, divided as “Normal” and “Mild or more” and ASSIST Risk Level, divided as Low Risk and Moderate/High Risk). Spearman’s correlation test was used to verify associations between quantitative variables. Descriptive statistics were carried out using Excel^®^, and all other inferential analyses were run with IBM^®^ SPSS Statistics software (v20) or SigmaStat^®^ 3.1. Differences and associations were considered statistically significant when P ≤.05. F values, t values, z values, as well as the n, confidence interval (CI) are presented next to P values. To assess the potential of false negatives, the model performs a power test according to the hypothesis test used. A β ≤ 0.2 was considered as an adequate value for tests in which the results presented a P value higher than.05. The sample size was calculated before the first study using the most prevalent drugs in the population as targets (alcohol, tobacco and cannabis), comparing the pre-pandemic versus pandemic alcohol use prevalence (main outcome).

## 3 Results

The survey reached a total of 95,184 Facebook^®^ users (link clicks: 1,613), along with individuals reached through email and WhatsApp^®^. A total of 3,348 participants started the questionnaire in the C1 phase (13). For this study, the total number of participants with valid answers in C1 corresponds to 2498. After email follow-ups, the number of respondents in C2 was 1045, and in C3, there were 969 responses; in total, the number of individuals who answered all three phases corresponds to 755 respondents. Not surprisingly, significant reductions in the absolute number of respondents were observed, precluding the analysis of some drug classes (such as opioids) and specific sociodemographic categories (Incomplete Secondary Education), except for alcohol, tobacco and cannabis, and low social distancing for cocaine), meaning there were not enough participants for the GLM analysis for some subgroups (sociodemographics). Observing the sociodemographic factors, detailed in **Table 1**, similarities appear between the original sample (13) and this cohort. For example, the prevalence of women was higher than men. Regarding marital status, the number of ‘Single’ respondents was the highest, followed by ‘Married’ or ‘in Stable union’. The most prevalent category for education was Complete Higher Education, followed by Incomplete Higher Education. Income was more evenly distributed, with half of the respondents reporting two or more minimum wages. As for the social distancing, High social distancing was informed by around 55% of the respondents during the C1 evaluation.

**Table 1:** Sociodemographic data of respondents which followed through with all three phases of data collection from 2020 to 2022.

Age (Mean ± SD)	33 (± 10,4)	18 - 68 a
Gender	*N*	*%*
Men	214	28,3
Women	534	70,8
Rather not answer	7	0,9
Marital Status		
Single/Divorced/Widowed	504	66,8
Married/Stable union	251	33,2
Education		
Incomplete Secondary Education	3	0,4
Complete Secondary Education	195	25,8
Higher Education or more	557	73,8
Income		
Up to R$ 750.00	42	5,6
From R$ 751.00 to 1,500.00	61	8,1
From R$ 1,501.00 to R$ 3,000.00	149	19,7
From R$ 3,001.00 to R$ 6,000.00	201	26,6
From R$ 6,001.00 toR$ 9,000.00	108	14,3
More than R$9,000.00	194	25,7
Social Distancing*		
Low	35	4,6
Medium	289	38,3
High	431	57,1
**TOTAL**	755	100%

*Social distancing categories were collapsed as follows: not doing social distancing and very flexible = “Low”, flexible and moderate = “Medium”, and rigorous and very rigorous = “High”.

**Figure 1:**
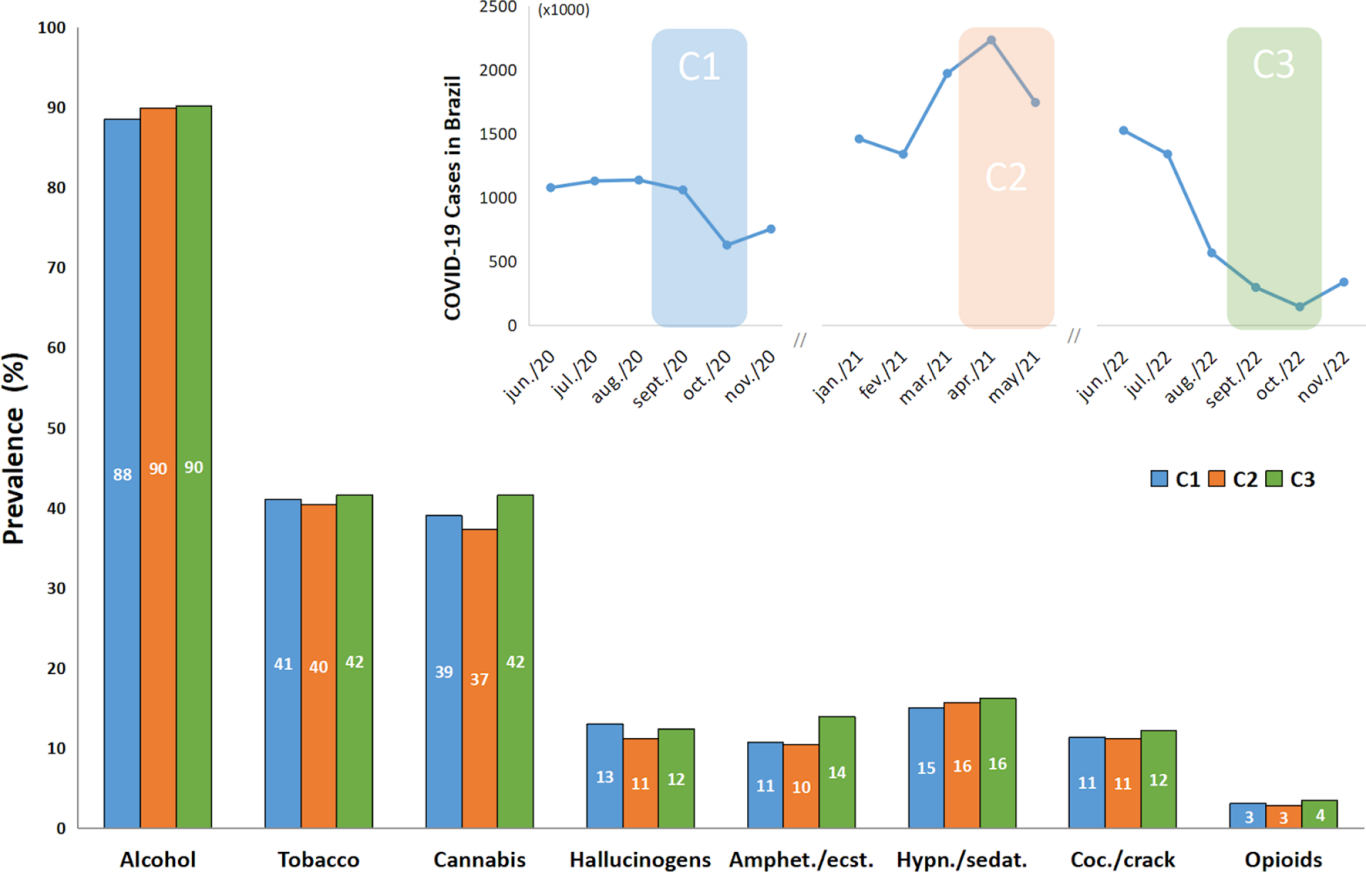
Prevalence of recent drug use according to ASSIST responses by time points (C1, C2 and C3) (n = 755). Top-right figure represents the number of cases associated with COVID-19 in Brazil monthly, and the colored shadow represents the data collection periods. Recent drug use in ASSIST refers to the use during the previous 3 months. The Chi-Square Test with the residual analysis when appropriate found no significant difference between time points (C1, C2 and C3). Amphet.”: amphetamines; “ecst.”: ecstasy; “Coc.”: cocaine; “Hypn.”: hypnotics; “sedat.”: sedatives.

The most prevalent drug used, considering the information of the absolute number of responses in the last three months of responding to the survey in all time points, is alcohol (around 90%), followed by tobacco and cannabis (approximately 40%), while the other drugs were all under 17% prevalence (**Figure 1**). For all drug classes, there was no significant difference between C1, C2 and C3, when analyzing the use prevalence. The graph at the top right in **Figure 1** represents the waves of cases caused by COVID-19 from June 2020 to November 2022 in Brazil and the C1, C2, and C3 data collection time points.

### 3.1 Assist score

**Figure 2:**
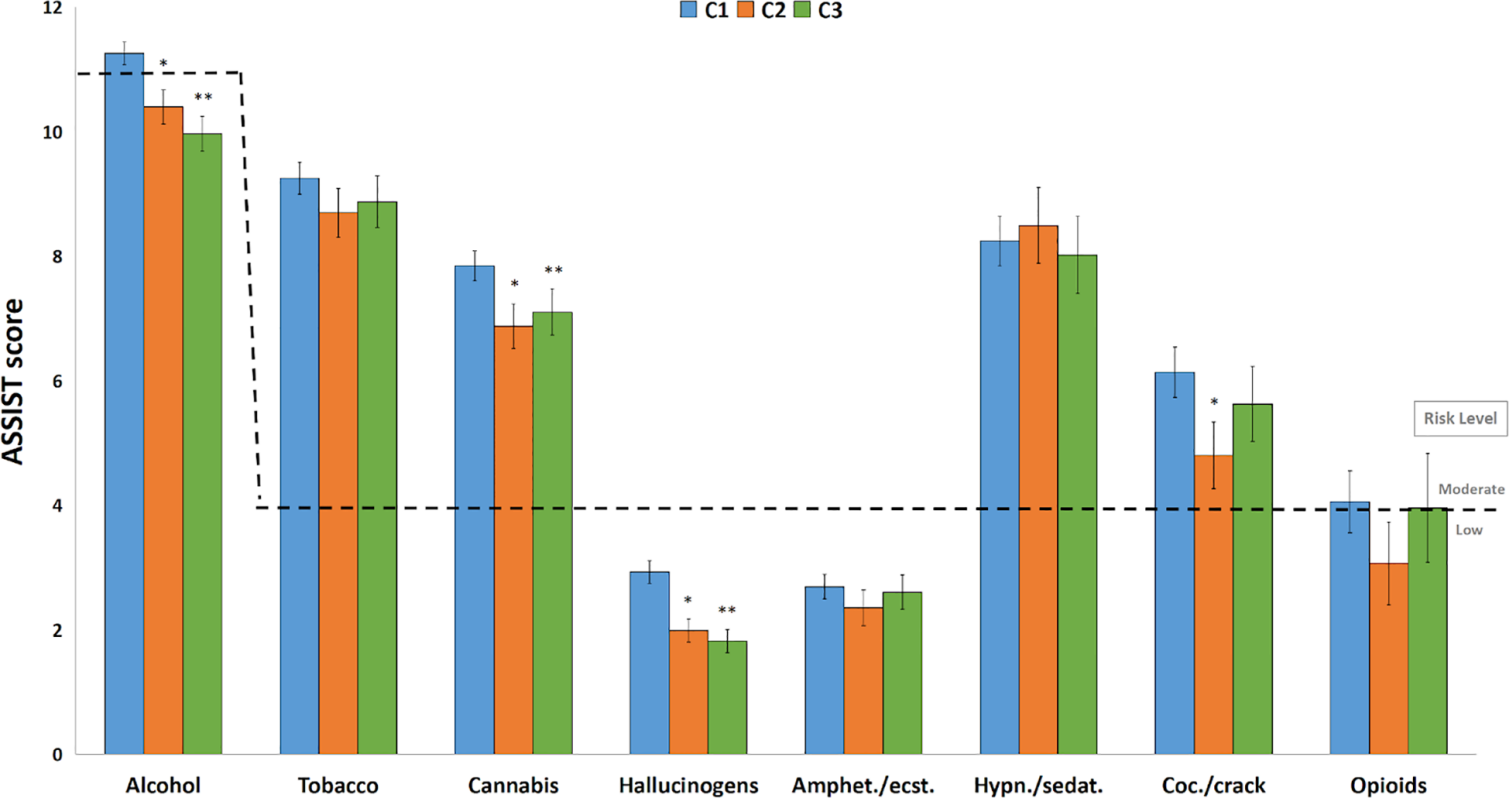
ASSIST average scores by time points. ASSIST scores (mean ± SEM) for each drug according to C1, C2 and C3 (n = 2498). Risk levels are represented in dashed lines according to ASSIST protocol. *: C2 lower than C1, *P* < 0.005; **: C3 lower than C1, *P* < 0.05, in the GLM. Amphet.”: amphetamines; “ecst.”: ecstasy; “Coc.”: cocaine; “Hypn.”: hypnotics; “sedat.”: sedatives.

The ASSIST mean scores are presented by time points, and the threshold between “Low Risk Level” and “Moderate Risk Level” is also shown in **Figure 2**. The “High Risk Level” is not shown in this figure because none of the mean scores were near its threshold. The highest scores besides alcohol, as we can see in **Figure 2**, are tobacco and cannabis, followed by hypnotics/sedatives and cocaine/crack. Overall, differences were observed between time points for some drugs, like alcohol, cannabis, hallucinogens and cocaine, when observing mean scores from the ASSIST scale. Differences were found between C1 and C2 and C1 and C3 for alcohol (P < 0.001), cannabis (P = 0.005), hallucinogens (P < 0.001) and cocaine (P < 0.05).

When assessed separately, the effect of time on mean scores for each drug class shows some interesting results. Alcohol mean scores demonstrated a progressive decrease over time, with C2 and C3 being lower than C1 (P < 0.005). Tobacco did not show statistically significant differences between time points, remaining at approximately the same level. Cannabis also presents differences and, although C3 has a slightly higher score than C2, both are lower than C1 (P < 0.05). Even though hypnotics/sedatives showed some greater scores in C1 and C2, it did not show statistically significant differences with C3, which almost fell to low risk level due to the high variability of the use during periods. Hallucinogens and Cocaine/crack presented only one difference between C1 and C2 (P = 0.035), as C3 showed a substantial decrease of score (**Figure 2**).

**Figure 3:**
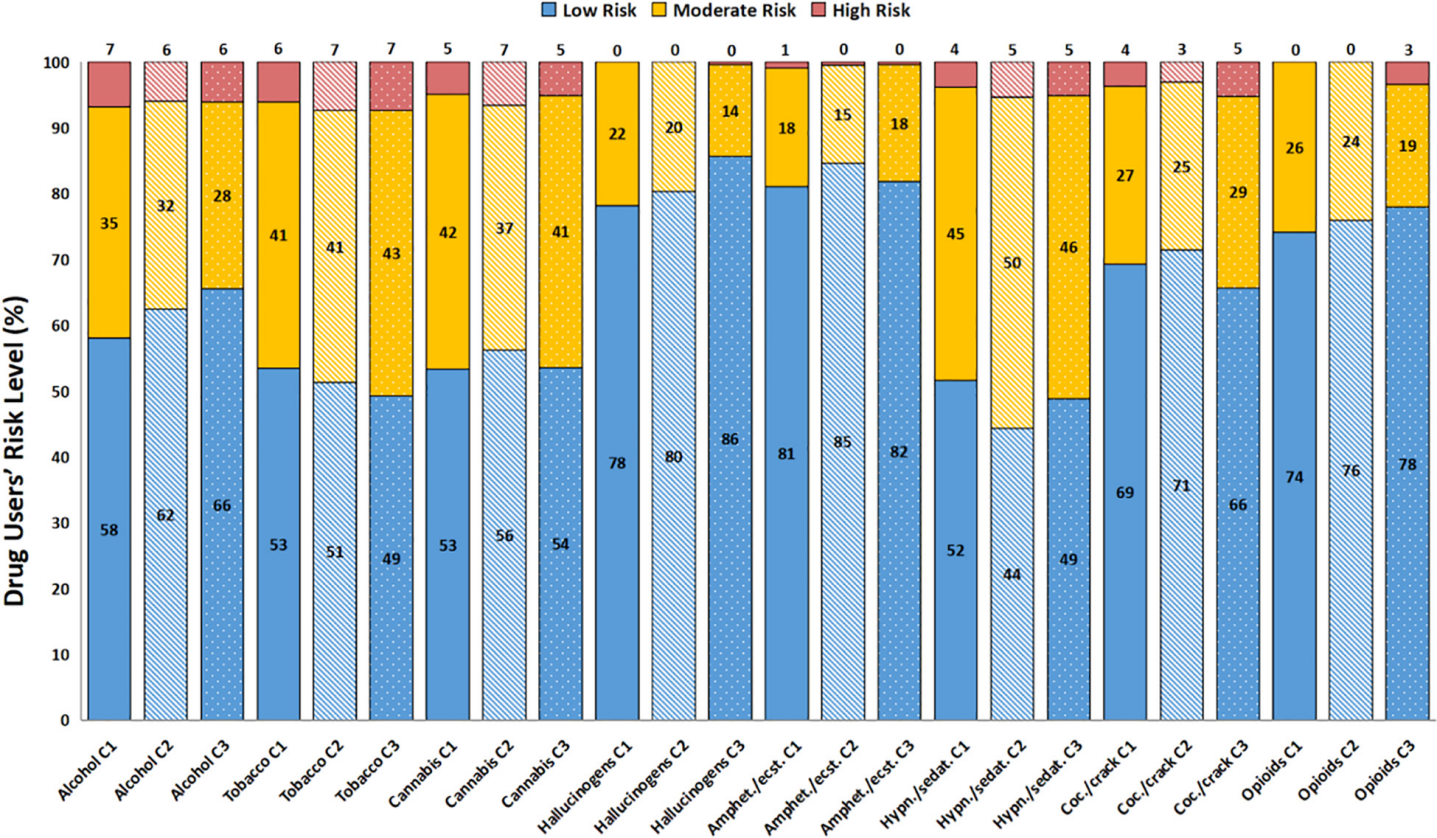
Drug users percentage and their respective ASSIST Risk Level (C1, C2 and C3). Drug user’s risk level is separated as low (blue columns), moderate (yellow stacked columns) and high (dark pink stacked columns). Time points are represented as different patterns: C1 - plain columns; C2 - stripped columns; C3 - dotted columns. The Chi-square test did not show differences between low and moderate/high risk level.

Regarding the ASSIST score risk level represented in dashed lines in **Figure 2**, it is observed that the mean scores for alcohol, tobacco, hypnotics/sedatives, and cocaine all reach the moderate risk level, with the exception of alcohol’s C2 (10.4 ± 0.6; CI95%: 9.7 – 10.9) and C3 (10.0 ± 0.6; CI95%: 9.3 – 10.5) frequencies and cocaine’s C2 frequency (4.8 ± 1.6; CI95%: 3.0 - 6.2), considering these CIs include the lower threshold of the moderate risk level, which is 11 for alcohol and 4 for the other drugs. The drug users’ risk level prevalence is presented in **Figure 3**. ASSIST risk levels showed higher prevalences in the low risk category. Moderate risk was mostly around 30%, and high risk was majorly lower than 10%. When comparing the low and moderate or more prevalences using the Chi-Square test, no statistically significant differences were found between groups.

The gender covariable has shown some statistically significant differences for several drug classes. Alcohol (P = 0.004), tobacco (P = 0.047), cannabis (P < 0.001), and hypnotics (P = 0.030) presented a gender effect, where men reported higher drug use than women. Only amphetamines/ecstasy demonstrated an interaction between the effect of the covariable and time (P = 0.011). Interestingly, for amphetamines/ecstasy, there were no statistically significant differences in the pairwise comparisons, only limitrophe non significance (Men’s C1 > Men’s C2; P = 0.053). This must be related to the pattern observed in their mean scores, since men showed a fall in C2 score, returning to a similar baseline score in C3, while women showed a rise in C2, and returned to a similar score to C1 in C3.

**Figure 4:**
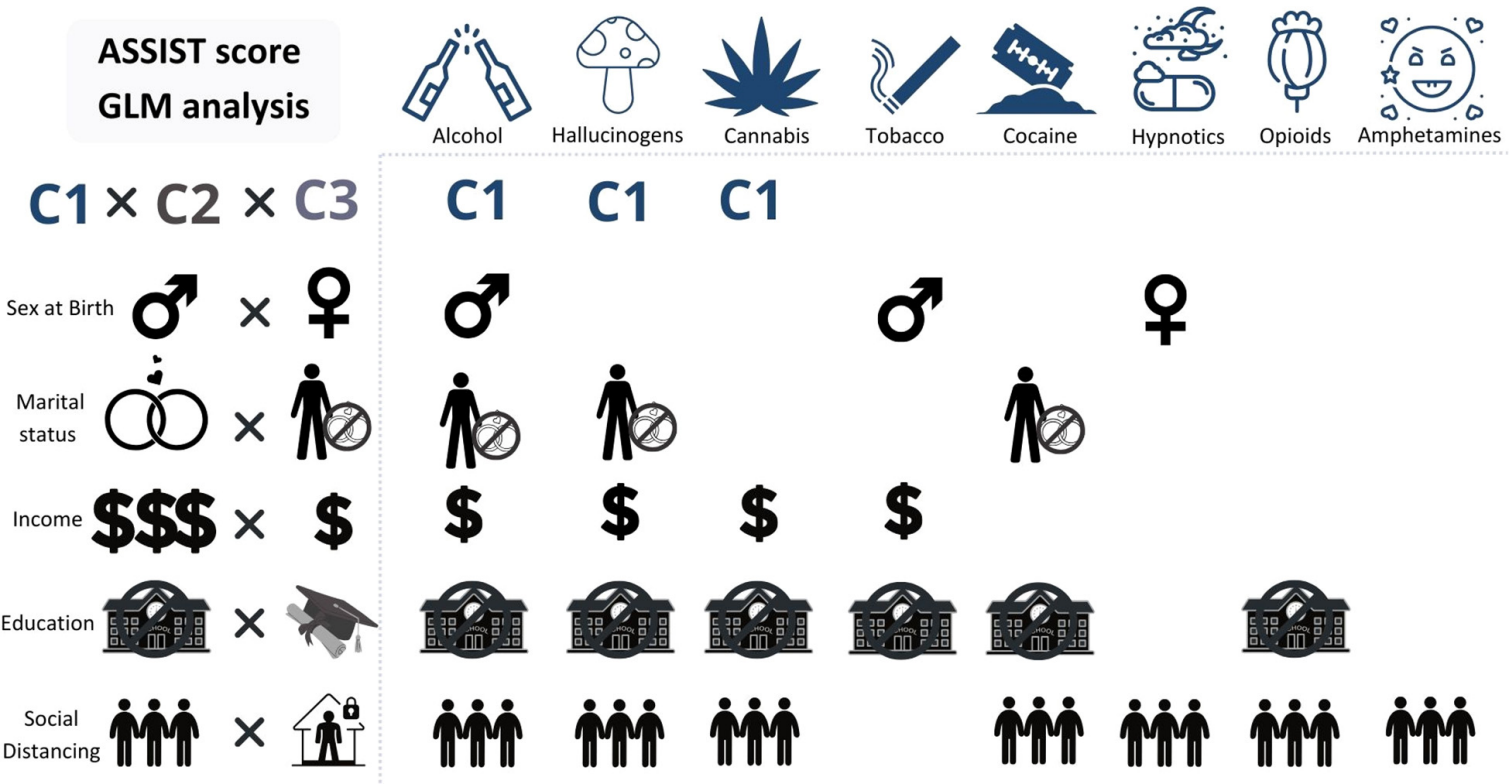
ASSIST score and sociodemographics by time points (C1, C2 and C3). GLM analysis of all three main variables. The “X” axis contains icons representing the drug classes, relative to the sociodemographic factors and their categories (“Y” axis) which showed statistical significant differences, meaning the sociodemographic factors presented under a drug class showed higher scores for that drug than the one it is compared to. “Y” Axis, from up to bottom: Time (C1 - C3): C1 had higher mean scores than the other time points for alcohol, hallucinogens, and cannabis. Gender (Men x Women): Men had higher mean scores for alcohol and tobacco; Women had higher mean scores for hypnotics/sedatives. Marital Status (Married/Stable union x Single/Divorced/Widowed): Single/Divorced/Widowed had higher mean scores for alcohol, hallucinogens, and cocaine. Income (Higher incomes x Lower incomes): Lower incomes showed higher mean scores for alcohol, hallucinogens, tobacco, and cannabis. Education (ISE x HE): almost all, except for hypnotics/sedatives and amphetamines/ecstasy. Social Distancing (Lower social distancing x Higher social distancing): almost all, except for tobacco. Icons in the “Y” axis: gender symbols for male and female. Married or stable union: rings; single/divorced/widowed: a person with a “no” symbol on top of rings. Income: higher than R$ 3,000.00, three dollar signs; lower than R$ 3,000.00, one dollar sign. Education: incomplete secondary education, a “no” symbol in front of a school; higher education, a trencher. Social distancing: lower social distancing, three people close together; higher social distancing, a person inside a house with a lock.

Another factor that showed an interaction between time and itself was “education”, again for amphetamines/ecstasy and also for hypnotics (P < 0.001). Although the majority of drug classes presented differences for covariable effects (all P < 0.001, except for hypnotics), alcohol, tobacco and cannabis were the only ones which did not present small sample sizes in C1 for Incomplete Secondary Education (ISE), while in C2 and C3, all drug classes presented small sample sizes for the mentioned category, hindering their analyses. Considering the most prevalent drug classes, Higher Education (HE) usually had lower scores than Secondary Education (SE). Amphetamines/ecstasy presented differences between SE and HE (SE in C1 > HE in all time points; SE in C2 > HE in C2; SE in C3 > HE in all time points). Tobacco, cannabis, hallucinogens, and cocaine users had higher scores for SE when compared to HE (P < 0.005), when considering only the covariable. Opioids only showed differences between ISE > SE, and ISE > HE (**Figure 4**, represented as lower education presenting a higher ASSIST score than higher education).

Income showed some statistical significant differences as well. An interaction effect was seen for alcohol users (P = 0.012) and hypnotics (P = 0.024), but also a time effect was seen for alcohol, hallucinogens (P < 0.001) and cocaine (P = 0.014) and a covariable effect for tobacco, cannabis (P < 0.001), hypnotics (P = 0.009), hallucinogens (P = 0.028). When we look at alcohol use and time points, we see that moderate to low incomes show higher scores than higher incomes (R$ 751.00 to 1,500.00 in C1 and C2, R$ 1,500.00 to 3,000.00 C1 > R$ 9,000.00 or more, C1 to C3; R$ 1,500.00 to 3,000.00, C1 > R$ 6,000.00 to 9,000.00, C1 and C2; R$ 1,500.00 to 3,000.00, all time points > R$ 6,000.00 to 9,000.00, C3). Hypnotics also showed an interaction between the covariable and time, and the comparisons by pairwise method showed differences between R$ 751.00 to 1,500.00 in C1 > R$ 6,000.00 to 9,000.00 in C2; R$ 3,001.00 to 6,000.00, C1 and C2 > R$ 6,000.00 to 9,000.00 C2; R$ 1,500.00 to 3,000.00, C1 and C2 > R$ 6,000.00 to 9,000.00, C2 (**Figure 4**, represented as lower income presenting a higher ASSIST score than higher income).

Social distancing showed some effects of the covariable and time. Alcohol use showed a covariable effect (P < 0.001) and a Time effect (P < 0.001). Cannabis use showed a covariable effect (P = 0.001) and a very close to significance time effect (P = 0.052). Amphetamines/ecstasy use also presented a covariable effect (P < 0.001). Hallucinogens presented a covariable and time effect (P < 0.001 and P = 0.001, respectively). Alcohol, cannabis, cocaine and hallucinogens showed differences as follows: lower social distancing greater than median social distancing and Lower social distancing greater than higher social distancing. Amphetamines/ecstasy had different patterns, with amphetamines/ecstasy showing one more difference (lower social distancing > median social distancing; lower > higher; median > higher, P < 0.05). Opioids only showed a difference between Low and Median (P < 0.05) (**Figure 4**, represented as lower social distancing presenting a higher ASSIST score than higher social distancing).

### 3.2 DASS-21

**Figure 5:**
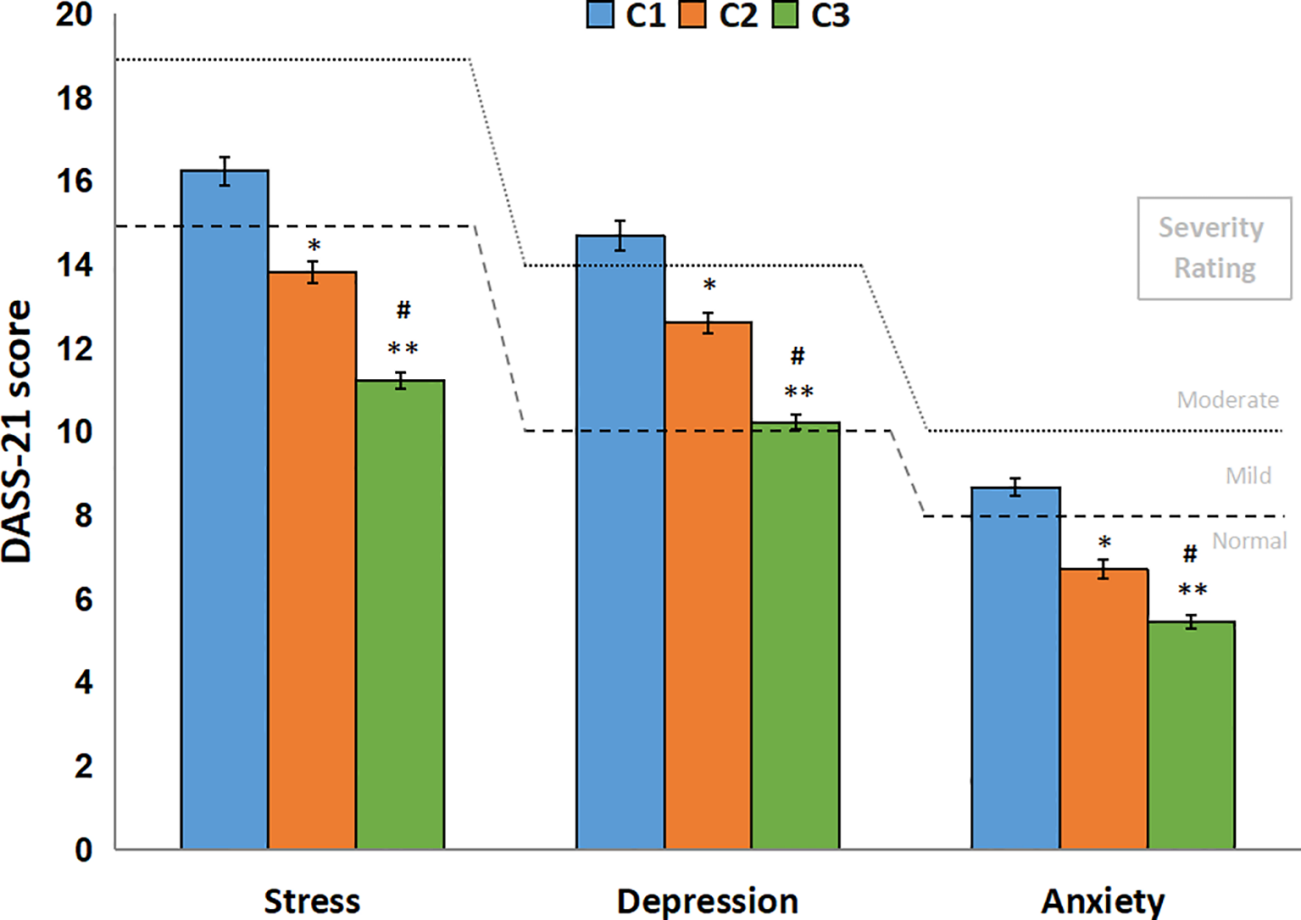
DASS-21 scores by time points (C1, C2 and C3). DASS-21 score (mean ± SEM) for all respondents in all time points (N = 755). Dashed lines represent DASS-21 severity rating for “mild” and dotted lines represent the severity rating for “moderate” of depression, anxiety and stress subscales. The generalized linear model showed differences between all time points for all three subscales. *: C2 lower than C1, *P* < 0.001; **: C3 lower than C1, *P* < 0.001; #: C3 lower than C2, *P* < 0.001.

Mean scores for depression, anxiety and stress traits mean scores are displayed in **Figure 5** and showed differences in all time points. For depression, mean scores were 14.70 (SEM: ± 0.27), 12.76 (SEM: ± 0.38) and 10.23 (SE: ± 0.36), for C1, C2 and C3, respectively. For anxiety, means were, also respectively, 8.68 (SEM: ± 0.21), 6.99 (SE: ± 0.28), 5.46 (SE: ± 0.24). And for stress, 16.24 (SEM: ± 0.24), 13.82 (SEM: ± 0.33), 11.22 (SEM: ± 0.32), also respectively. Considering the DASS-21 scale cutoffs, it can be observed that the C1 mean score for depression is in the moderate rating, while the others are in the mild rating. C2 mean scores are in the borderline between mild and normal ratings, and lastly, C3 mean scores are in the Normal range, except for the depression mean score.

**Figure 6:**
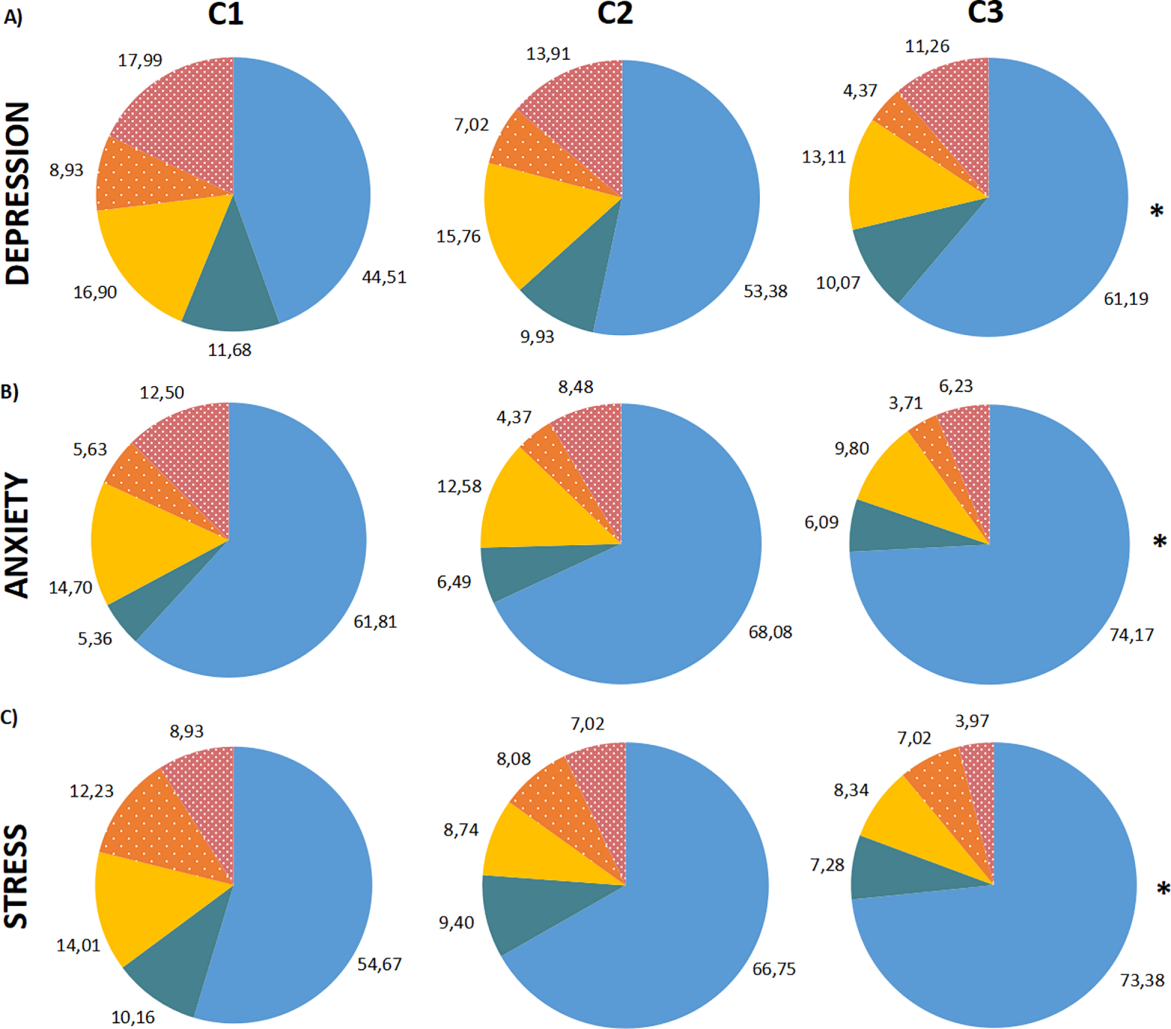
DASS-21 severity ratings in percentages (%) by time points (C1, C2 and C3). Percentage of the sample distributed in the subscale ratings. **(A)** Depression subscale, in order of time points. **(B)** Anxiety subscale, in order of time points. **(C)** Stress subscale, in order of time points. The severity ratings are colored as “Normal”: blue; “Mild”: dark green; “Moderate”: yellow; “Severe”: dotted orange; “Extremely severe”: dotted dark pink. The Chi-Square Test was performed comparing “Normal” and “Mild or more” categories. *: P < 0.0001, “Normal” in C3 higher than in C1.

DASS-21 prevalences from the DASS-21 scale are presented in **Figure 6**. In view of the prevalences observed for each time point, a few interesting considerations can be taken. The most extreme categories (“normal” and “extremely severe”) were the ones which changed the most from C1 to C3. The depression scale suffered increases in the “normal” category (44.51% to 61.19%), while the “extremely severe” suffered reductions (17.99% to 10.61%). “mild” and “moderate” reduced slightly (approximately -2% for each time point), and “severe” barely reduced percentages. Anxiety scale also suffered similar changes in percentages, although “normal” was the only one showing increases (66.07% to 74.17%). “mild” category showed an increase in C2, but reduced again in C3. “moderate”, “severe”, and “extremely severe” changed approximately -3% for each time point, suggesting a normalization of symptoms. Stress scale exhibited similar changes to anxiety. The only category with raises in percentages was “normal”, the rest decreased around 3 to 4% (“moderate” to “extremely severe” falling almost in half in C3). A chi-square test was run for DASS-21 ratings prevalences, comparing normal and mild to extremely severe ratings, showing statistically significant differences for all three subscales, indicating C1 had lower frequency than expected and C3 had more than expected.

**Figure 7:**
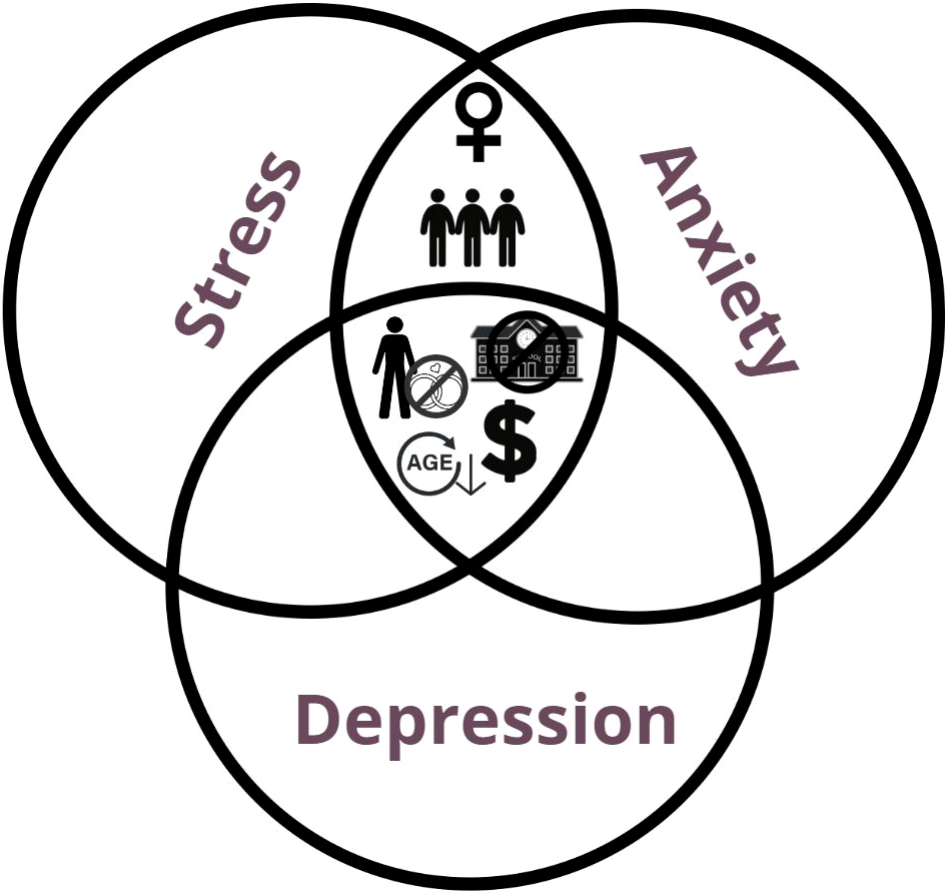
DASS-21 score and sociodemographic factors. GLM analysis for DASS-21 scores and Sociodemographic factors. Depression, Stress and Anxiety subscales were separated for a better visualization. The icons positioned around each subscale are relative to the sociodemographic factors and their categories which showed statistical significant differences, meaning the sociodemographic factors presented showed higher scores for that subscale than the one it is compared to. Gender (Men x Women): Women had higher mean scores for anxiety and stress (P < 0.001). Marital Status (Married/Stable union x Single/Divorced/Widowed): Single/Divorced/Widowed had higher mean scores for all three subscales (Depression, interaction: P < 0.05; Anxiety, interaction: P = 0.002; Stress, interaction: P < 0.001). Income (Higher than R$ 3,000.00 incomes x Lower than R$ 3,000.00 incomes): Lower incomes showed higher mean scores for all three subscales (Depression: P < 0.001; Anxiety: P < 0.001; Stress: P < 0.001). Education (ISE x HE): ISE showed higher mean scores for all three subscales (Depression: P = 0.002; Anxiety: P = 0.002; Stress: (P < 0.05). Social Distancing (Lower social distancing x Higher social distancing): Lower social distancing showed higher mean scores for stress (P = 0.015) and anxiety (P = 0.027). The icons used are the same as used in **Figure 4**. Icons in the “Y” axis: gender symbols for male and female. Married or stable union: rings; single/divorced/widowed: a person with a “no” symbol on top of rings. Income: higher than R$ 3,000.00, three dollar signs; lower than R$ 3,000.00, one dollar sign. Education: incomplete secondary education, a “no” symbol in front of a school; higher education, a trencher. Social distancing: lower social distancing, three people close together; higher social distancing, a person inside a house with a lock.

All three traits in C1 exhibited higher mean scores than C2 and C3, while C2 was higher than C3 (P < 0.001) (**Figure 7**, represented as dichotomous variables). As for the sociodemographic factors, when analyzed through the Two-Way RMAnova, marital status exhibited an interaction with time for all subscales (Two-Way RMAnova; P < 0.05). Single/divorced/widowed presented greater scores for depression, anxiety and stress than married/stable union (P < 0.001). Gender demonstrated a covariable effect for anxiety and stress subscales (P < 0.001) and the scores were greater for women. Education level presented associations with depression, anxiety and stress. HE showed lower scores of depression (P = 0.002) and stress (P = 0.027). For income, stress and anxiety showed higher scores for the R$ 1,501 to 3,000 and R$ 3,001 to 6,000 ranges than higher incomes (more than R$ 6,001), especially in C2 and C3. Also, social distancing presented higher scores of anxiety and stress for lower social distancing when compared to medium and high social distancing (P < 0.05).

The Spearman’s correlation demonstrated positive correlations between ASSIST scores and DASS-21 scores for all subscales and drugs (**Supplementary Table S1**). However, r coefficients hardly surpassed the 0.20 mark, which indicates rather weak associations, taking in account that the P-values were statistically significant. The strongest correlations were found for opioids and hypnotics (around 0.30-0.40), especially in C1 and C2. Amphetamines/ecstasy showed somewhat stronger correlations in C2 (around 0.30) as well, but not in other time points. Only the correlation between Cocaine/crack versus stress was not significant (P = 0.116). This means both scores are somehow linked, although not very strongly, and might be influencing one another, as both scores suffered reductions through time.

## 4 Discussion

This study aimed to investigate the impact of the pandemic on substance use patterns and psychological health of Brazilians. To address this, we assessed how the three years of pandemic could affect drug use habits in a Brazilian population and if changes could be associated with mood state symptoms and/or sociodemographic factors. Despite no changes were observed in the prevalence of use of all drugs, during the three time points investigated, ASSIST scores did not show the same pattern. Alcohol, cannabis, hallucinogens and cocaine/crack users reported a decrease in ASSIST scores in C2 (during a pandemic peak) compared to the C1 (during the beginning of pandemic), which was maintained during C3 (already decreed the end of national health emergency of the pandemic in Brazil), except for cocaine/crack. This might be explained by the emotional state of this sample, possibly related to fear of the pandemic. This aligns with the fact that C2 data were collected shortly after a significant surge COVID-19 cases and deaths, accompanied by stricter contingency measures, such as curfews, restrictions on non essential activities restrictions and circulation of people, among other measures implemented nationwide (23). In contrast, by the time of C3, approximately 84% of the Brazilian population had received at least one dose of the COVID-19 vaccine (C2: 23%, C1: 0%) (24), which may have alleviated pandemic-related fear and contributed to changes in the observed patterns. These findings suggest that pandemic policies, including vaccination and social distancing measures, may have directly influenced behavioral trends over time.

This could also be related to resilience to the pandemic after nearly two years. Studies (11, 25) have shown that participants who reported functional social support exhibited fewer symptoms of anxiety or depression and lower drug use (11, 25). A range of psychobiological mechanisms may underlie the relationship between resilience to stress and drug use. For instance, stress or adversity can alter certain functions of the mesolimbic pathway, increasing vulnerability to drug use (26–28). Social determinants, such as positive social interactions and social support, also play a critical role in fostering resilience. Positive social interactions can encourage adaptive coping strategies (29, 30), whereas insufficient or “negative” social support can contribute to maladaptive coping mechanisms, including risk-taking behaviors and drug use (26, 31).

Another factor potentially contributing to a sense of security during the pandemic was the implementation of vaccination programs (32, 33), which began in Brazil in 2021. With relatively low vaccine hesitancy among Brazilians (34), the reduction in COVID-19 cases and deaths led to the relaxation of contingency measures. Reduced isolation may have influenced the relationship between substance use and mental health, possibly mediated by vaccination rates. For instance, as observed in our previous study (13), higher levels of social distancing were associated with a lower percentage of drug use, suggesting that limited exposure to substances and heightened concern over the consequences of seeking or using drugs may have played a role.

However, different results are observed in other countries, like in Australia and the US (25, 35). The National Wastewater Drug Monitoring Program of Australia found that alcohol, cocaine and heroin levels in water had a decrease between April and August of 2022, while cannabis, oxycodone, fentanyl and MDMA levels showed an increase (25). The 2022 National Survey on Drug Use and Health reported a very slight increase from 2021 to 2022 of the following drugs: alcohol, cannabis, cocaine, meth, tranquilizers or sedatives and opioids. Tobacco showed a decrease, and hallucinogens did not change (35). In the present study we did not find the same chronological pattern compared to those ones, but the mitigation and timelapse of this sanitary phenomenon was not completely comparable.

Conversely, ASSIST also presents an intervention by the end of the questionnaire, showing which risk level the respondent is included in, which might have introduced a bias regarding reduced drug use scores. Although the brief intervention was tested and validated using a randomized controlled trial in four different countries (Brazil, India, Australia and USA) (36), their results showed that even the controls, who did not receive an intervention, had reductions in scores. Thus, the plain application of a questionnaire could present an effect in drug use score, which could happen in other studies as well, considering that the exposure to a survey on drug use could bring awareness to drug use and make respondents rethink their habits.

It is relevant to note that our sample seems to differ somewhat from other studies performed at the national level in Brazil. The most recent studies that report heavy episodic drinking and tobacco use reported in the last month (meaning between September of 2021 and February of 2022, and between December of 2022 and April of 2023) show an increasing trend of heavy episodic drinking and smoking (37, 38). Among heavy episodic drinkers and smokers, men were more prevalent than women, and both had an increase from one year to the other. Education years were different for alcohol and tobacco users. Drinkers had a higher number of education years than smokers, which is opposite from our results (37, 38). In our sample, higher education is more prevalent in alcohol and tobacco users. This may be related to the emotional state of young adults who are pursuing undergraduate degrees, as they have high levels of stress and depression symptoms, loneliness and worry feelings, as we can observe in studies with this population (10). Another aspect is that marital status has shown a relationship with the ASSIST scores for some drugs. A Japanese study found results that come in line with this, showing single people were more likely to perceive alcohol consumption and unhealthy lifestyle (39).

A study in a Canadian sample of people who use drugs (data collected between the second half of 2020 and the beginning of 2021) found that around 39% of their sample reported a decrease in income since the onset of the pandemic, and they also reported an overall drug use increase for drinking, use of cigarettes, e-cigarettes and cannabis (40). This comes in agreement with our results regarding income. Another study (32) from the USA reported that loss of income was among reasons for a decrease in drug use, as well as other stressful factors, such as social isolation and staying home. It must be highlighted that in the present research - and also one those ones presented, it is not clear if the reduction in income could be the cause or consequence of drug use and/or with, for instance, the limited social interaction due to the pandemic.

The DASS-21 severity ratings demonstrate a reduction of scores from C1 to C3. One hypothesis that could explain this phenomenon is that the population became gradually more accustomed to the context of the pandemic, which somewhat subsided symptoms related to their emotional state. Another hypothesis could be due to the relaxation of contingency measures. The increase in prevalence of people reporting more normal ratings for the three subscales further supports these inferences. A few cohort studies showed that anxiety levels were associated with higher risk of drug misuse (41), while depressive symptoms and loneliness were associated with binge drinking and functional social support was inversely associated with recreational drug use (11). If we compare these findings to our sample, for which a decrease in ASSIST for some drugs and DASS-21 scores was observed through time, we might infer that their emotional state has influenced their use, although we cannot pinpoint the cause to more resilience or more social support.

If we compare these findings to our sample, where a decrease in ASSIST scores for some drugs and DASS-21 scores was observed over time, we might infer that their emotional state influenced their substance use. However, it is difficult to attribute this to increased resilience or enhanced social support without deeper insights. Incorporating qualitative data, such as interviews or open-ended surveys, could provide a richer understanding of individual coping mechanisms and the role of community support systems in shaping these trends. Such data could help clarify how participants navigated stressors and whether specific protective factors contributed to the observed changes.

In our study, depression, anxiety and stress were associated with younger age, single/divorced/widowed, lower income, and lower education, moreover, women and lower social distancing with anxiety and stress. For this population, education and income had an effect mainly on anxiety and stress scores. In general terms, the greater the education and income levels, the lower the stress, anxiety and depressive symptoms, indicating a protective role of higher degrees of education and income. The results are similar to a study by Calegaro’s et al. (42), where the authors report an association between symptoms of anxiety and low levels of education. A discrepancy was found in another study (43) that discussed the probable presence of health anxiety, as higher education levels were related to the psychological symptoms. Another similar result is the gender effect, in which Calegaro et al. (42) reported higher levels of all subscales whereas we report higher levels of anxiety and stress in women (42). On this note, the literature shows that women have some psychological and socioeconomic traits which make them more prone to internalizing mental health disorders (44). A study investigating the risk factors for depression and distress (45) found that being single or widowed and living alone were linked to both symptoms, which comes in line with our data.

An interesting approach is that the most stressful and anxiogenic period of the pandemic (between March and May of 2020) (46) was not included in our first time point, as data collection was performed in September-October of 2020. Nevertheless, it is also perceived in the literature that stressful or harmful events may lead to depression later on (22, 47). With the first report, we might have seen a subchronic effect of the worst period of the pandemic, with moderate levels of depressive symptoms, while stress and anxiety were already waning.

The Spearman’s correlation could be a useful tool to confirm the relationship of the ASSIST scores and DASS-21 scores as both were progressively reduced through time points, which suggests the amelioration of symptoms. Though coefficients were not very strong, alcohol, tobacco, hypnotics, and opioids showed a few stronger coefficients through C1 to C3, indicating these users might be more vulnerable to stressful events. A cross-sectional study that was also conducted in Brazil (42) used a different approach, in which its focus was directed to the DASS-21 scale and PTSD symptoms. They also assessed the results of the DASS-21 and the link with the presence of drug use, for which alcohol, tobacco, cocaine, ecstasy or LSD, benzodiazepines and opioids users prevalence was much lower than in our sample. They reported an association between depression and PTSD symptoms with alcohol consumption, use of tobacco, cannabis and benzodiazepines, and stress with alcohol consumption and use of benzodiazepines.

In view of some populations strata presenting some changes in different drug classes, such information is important so the governmental organs can plan in a more adequate manner to mitigate the impact of drug use, as it is advocated by the National Plan of Policies on Drugs, previewed in the Law n° 13,840/2019 (48). This National Plan recognizes the importance of preventing drug use and implementing measures and programs to reduce the hazardous consequences of drug use and abuse, as much as enhancing healthcare, social assistance and promoting better access to public services. Therefore, there might emerge a need to pursue other routes not explored by this study, e.g., when considering the complexities of polydrug use and novel psychoactive substances (NPS) use screening and therapies, which could have a range of implications and challenges (14), which are relevant in the current context. NPS detain a whole level of complications for its detection and consequences to the organism, in view of their rapid evolution, variability in composition, and limited availability of reliable detection methods, complicating both screening and law enforcement efforts (49, 50).

Additionally, they can be much stronger or unpredictable in its effects (51), and even more, be used in conjunction with other drugs, raising the fatality risk, such as the case of fentanyl analogues, which are able to provoke a strong respiratory depression and deaths (52). Polydrug use amplifies risks by increasing the likelihood of adverse drug interactions, complicating clinical management, and contributing to worse mental health outcomes (53). Many overdose deaths are caused by the adulteration of street drugs with other drugs or psychoactive substances, such is the case with cocaine or methamphetamine and lacing with substances like fentanyl, ketamine, levamisole, and others (54–56). Adulterants further exacerbate these risks, as their presence in illicit drugs can lead to severe health consequences and pose significant hurdles for public health initiatives. And not only that, therapy strategies can be strongly impaired if polydrug use is not even considered or known, as metabolites can remain on the body for some time and likely affect recovery/relapse (57).

Evidence-based guidelines addressing these issues, as seen in countries with advanced drug monitoring systems, highlight the need for more robust surveillance, public education campaigns, and targeted interventions (58–60). Strengthening the capacity to detect and respond to these emerging threats should be a priority within national strategies, including this National Plan, to ensure the development of effective and adaptive measures for drug use prevention and harm reduction.

### 4.1 Limitations

One important thing to note is that the sample was obtained from an online survey, disclosed and spread through social media (Facebook^®^ and Instagram^®^), message apps and emails. This might introduce a selection bias, as this constitutes a convenience sample, which may lead to impaired internal validity.

As could be seen from the sample size we obtained from this 3-year cohort, there was an unsurprisingly significant participant dropout rate (41). It is important to note that prospective studies are always prone to loss of follow up from participants (61), as they may not feel as engaged or motivated to participate in the study as they were in the beginning. This is especially true when taking into account how diverse were the COVID-19 waves and prospects during 2022 when compared to 2020. Nonetheless, this shortage in sample size did not hinder statistical analyses completely, as the model used still had enough power to perform several comparisons between various subgroups. There is a loss of follow-up rate for all drug classes when we observe absolute numbers. However, they maintain proportional numbers when percentages are calculated from the total number of each time point. This indicates no biased loss for one or more drug classes or in one group. Although use of inhalants was excluded from our results, we believe this had little impact in our findings due to the remarkably low prevalence of abuse for this particular class of substances in Brazil.

An aspect of the GLM is that it makes analyses of data in a manner that enables the comparison of time points. Some participants have answered all 3 segments of the cohort, while some only answered the first and the second sequels, or the first and the third ones. The model is designed to handle varying types of error distributions and varying sample sizes along remitted measures.

Drug-specific questions from the ASSIST questionnaire required information about their drug use in the last three months. This introduces a memory bias (61) because, even if this period of time is not considered to be that extensive, some people might have more difficulty in remembering their past drug use pattern than others. Importantly, C2 and C3 participants were asked to make this assessment years later. However, they were most likely motivated to pay attention to their own habits due to media’s constant disclosure of mental health issues emerging during the pandemic, as well as to the fact that they were being “observed” by researchers from this study, making them less prone to forget their drug use and mood state symptoms. Nevertheless, this study highlights the dynamic nature of drug use and emotional well-being during prolonged periods of social disruption, emphasizing the need for targeted public health strategies to support at-risk populations during such crises.

### 4.2 Conclusions

Through this cohort, it was possible to address a great mental health issue very much enlivened in the pandemic, which was the extension of the impact of the pandemic on drug use and emotional state, in the first year of the pandemic when very strict physical distancing measures took place and how it might have changed in subsequent years (more or less flexible physical distancing measures).

In conclusion, drug use for several drugs presented a reduction in scores when comparing the time points soon after the end of the pandemic with its beginning, and the same happened to the emotional state symptoms, implying a development of resilience or an accommodation to the context. An important aspect to note is that sociodemographic strata showed different drug use patterns and emotional states. Men, single/divorced/widowed, lower education, income and social distancing respondents perceived more drug use score, which comes in line with the literature. Finally, women, younger age, single/divorced/widowed, lower education, income and social distancing showed higher scores for the emotional/mood state.

Altogether, these insights are important for comprehending what steps can be taken for future research in drug use and abuse/mental health of similar populations. Even though the general Brazilian population might be much more diverse than our sample, our findings still provide a valid source of insights for a great part of the population. By acknowledging the dynamic relationship between drug use and emotional well-being, policymakers and healthcare providers can develop targeted interventions that address the unique challenges faced by at-risk populations, ultimately promoting resilience and improving overall well-being during times of crisis.

## Data Availability

The raw data supporting the conclusions of this article will be made available by the authors, without undue reservation.

## References

[1] Mathieu E Ritchie H Rodés-Guirao L Appel C Giattino C Hasell J. Coronavirus Pandemic (COVID-19). Our World Data. (2020). doi: 10.1038/s41562-021-01122-8

[2] Sarker R Roknuzzaman ASM Nazmunnahar Shahriar M Hossain M Islam M. The WHO has declared the end of pandemic phase of COVID-19: Way to come back in the normal life. Health Sci Rep. (2023) 6:e1544. doi: 10.1002/hsr2.1544 37674622 PMC10478644

[3] Croda J Oliveira WKD Frutuoso RL Mandetta LH Baia-da-Silva DC Brito-Sousa JD. COVID-19 in Brazil: advantages of a socialized unified health system and preparation to contain cases. Rev Soc Bras Med Trop. (2020) 53:e20200167. doi: 10.1590/0037-8682-0167-2020 32320998 PMC7182282

[4] Marel C Mills KL Teesson M. Substance use, mental disorders and COVID-19: a volatile mix. Curr Opin Psychiatry. (2021) 34:351–6. doi: 10.1097/YCO.0000000000000707 PMC8183242 33741762

[5] Dos Santos CF Picó-Pérez M Morgado P. COVID-19 and Mental Health—What Do We Know So Far? Front Psychiatry. (2020) 11:565698. doi: 10.3389/fpsyt.2020.565698 33192687 PMC7649114

[6] Schmidt RA Genois R Jin J Vigo D Rehm J Rush B. The early impact of COVID-19 on the incidence, prevalence, and severity of alcohol use and other drugs: A systematic review. Drug Alcohol Depend. (2021) 228:109065. doi: 10.1016/j.drugalcdep.2021.109065 34600257 PMC8455354

[7] Huang Y Zhao N. Generalized anxiety disorder, depressive symptoms and sleep quality during COVID-19 outbreak in China: a web-based cross-sectional survey. Psychiatry Res. (2020) 288:112954. doi: 10.1016/j.psychres.2020.112954 32325383 PMC7152913

[8] Volkow ND. Collision of the COVID-19 and Addiction Epidemics. Ann Intern Med. (2020) 173:61–2. doi: 10.7326/M20-1212 PMC7138334 32240293

[9] Vai B Mazza MG Delli Colli C Foiselle M Allen B Benedetti F. Mental disorders and risk of COVID-19-related mortality, hospitalisation, and intensive care unit admission: a systematic review and meta-analysis. Lancet Psychiatry. (2021) 8:797–812. doi: 10.1016/S2215-0366(21)00232-7 34274033 PMC8285121

[10] Remesan AK Sekaran VC Jothikaran TAJ Ashok L. Substance Use among Emerging Adults during the COVID-19 Pandemic: A Review through the Lens of Sustainable Development Goals. Int J Environ Res Public Health. (2023) 20:6834. doi: 10.3390/ijerph20196834 37835104 PMC10572374

[11] Meanley S Choi SK Thompson AB Meyers JL D’Souza G Adimora AA. Short-term binge drinking, marijuana, and recreational drug use trajectories in a prospective cohort of people living with HIV at the start of COVID-19 mitigation efforts in the United States. Drug Alcohol Depend. (2022) 231:109233. doi: 10.1016/j.drugalcdep.2021.109233 34998247 PMC8709730

[12] Horigian VE Schmidt RD Feaster DJ. Loneliness, Mental Health, and Substance Use among US Young Adults during COVID-19. J Psychoactive Drugs. (2021) 53:1–9. doi: 10.1080/02791072.2020.1836435 33111650

[13] Nin MS Heidrich N Almeida FB Izolan LR Constant HMRM Freese L. Social distancing and changes in drug use: Results from a cross-sectional study during the COVID-19 pandemic in Brazil. Front Psychiatry. (2022) 13:999372/full. doi: 10.3389/fpsyt.2022.999372/full 36440408 PMC9682187

[14] Di Trana A Carlier J Berretta P Zaami S Ricci G. Consequences of COVID-19 Lockdown on the Misuse and Marketing of Addictive Substances and New Psychoactive Substances. Front Psychiatry. (2020) 11:584462/full. doi: 10.3389/fpsyt.2020.584462/full 33192730 PMC7644618

[15] Jabalameli MR Zhang ZD. Substance abuse and the risk of severe COVID-19: Mendelian randomization confirms the causal role of opioids but hints a negative causal effect for cannabinoids. Front Genet. (2022) 13:1070428. doi: 10.3389/fgene.2022.1070428 36583016 PMC9792508

[16] Wang QQ Kaelber DC Xu R Volkow ND. COVID-19 risk and outcomes in patients with substance use disorders: analyses from electronic health records in the United States. Mol Psychiatry. (2021) 26:30–9. doi: 10.1038/s41380-020-00880-7 PMC7488216 32929211

[17] Dubey MJ Ghosh R Chatterjee S Biswas P Chatterjee S Dubey S. COVID-19 and addiction. Diabetes Metab Syndr. (2020) 14:817–23. doi: 10.1016/j.dsx.2020.06.008 PMC7282772 32540735

[18] Humeniuk R Ali R Babor TF Farrell M Formigoni ML Jittiwutikarn J. Validation of the Alcohol, Smoking And Substance Involvement Screening Test (ASSIST). Addict Abingdon Engl. (2008) 103:1039–47. doi: 10.1111/j.1360-0443.2007.02114.x 18373724

[19] Schrempft S Pullen N Baysson H Zaballa ME Lamour J Lorthe E. Mental health trajectories among the general population and higher-risk groups following the COVID-19 pandemic in Switzerland, 2021–2023. J Affect Disord. (2024) 359:277–86. doi: 10.1016/j.jad.2024.05.065 38772508

[20] Hu K Hu Y Godfrey K Li Q Li CSR. A 2-year mental health follow-up study subsequent to COVID-19. Psychiatry Res. (2024) 333:115684. doi: 10.1016/j.psychres.2023.115684 38219344

[21] Henrique IFS De Micheli D Lacerda RBD Lacerda LAD Formigoni MLODS. Validação da versão brasileira do teste de triagem do envolvimento com álcool, cigarro e outras substâncias (ASSIST). Rev Assoc Médica Bras. (2004) 50:199–206. doi: 10.1590/S0104-42302004000200039 15286871

[22] Vignola RCB Tucci AM. Adaptation and validation of the depression, anxiety and stress scale (DASS) to Brazilian Portuguese. J Affect Disord. (2014) 155:104–9. doi: 10.1016/j.jad.2013.10.031 24238871

[23] Tokarnia M Valente J Cruz E. Agência Brasil. Covid-19: com aumento de mortes, estados reforçam restrições (2021). Available at: https://agenciabrasil.ebc.com.br/geral/noticia/2021-03/Covid-19-com-aumento-de-mortes-estados-reforcam-restricoes (Accessed December 10, 2024).

[24] Departamento de Monitoramento, Avaliação e Disseminação de Informações Estratégicas em Saúde (DEMAS) Secretaria de Informação e Saúde Digital (SEIDIGI) Ministério da Saúde. gov.br. Vacinometro COVID-19 (2024). Available online at: https://infoms.saude.gov.br/extensions/SEIDIGI_DEMAS_Vacina_C19/SEIDIGI_DEMAS_Vacina_C19.html (Accessed July 11, 2024).

[25] Tscharke B O’Brien J Bade R Prasad P Barry D Elisei G. National Wastewater Drug Monitoring Program. Canberra City: Australian Criminal Intelligence Commission (2023). p. 94. Available at: https://www.acic.gov.au/publications/national-wastewater-drug-monitoring-program-reports. Report No.: 19 (Accessed July 25, 2024).

[26] Volkow ND Blanco C. Substance use disorders: a comprehensive update of classification, epidemiology, neurobiology, clinical aspects, treatment and prevention. World Psychiatry. (2023) 22:203–29. doi: 10.1002/wps.21073 PMC10168177 37159360

[27] Jouhki H Oksanen A. To Get High or to Get Out? Examining the Link between Addictive Behaviors and Escapism. Subst Use Misuse. (2022) 57:202–11. doi: 10.1080/10826084.2021.2002897 34809536

[28] al’Absi M. The influence of stress and early life adversity on addiction: Psychobiological mechanisms of risk and resilience. Int Rev Neurobiol. (2020) 152:71–100. doi: 10.1016/bs.irn.2020.03.012 32451001 PMC9188362

[29] El Rawas R Amaral IM Hofer A. Social interaction reward: A resilience approach to overcome vulnerability to drugs of abuse. Eur Neuropsychopharmacol. (2020) 37:12–28. doi: 10.1016/j.euroneuro.2020.06.008 32624295

[30] Hassanbeigi A Askari J Hassanbeigi D Pourmovahed Z. The Relationship between Stress and Addiction. Proc - Soc Behav Sci. (2013) 84:1333–40. doi: 10.1016/j.sbspro.2013.06.752

[31] Oakland AP. Avoidance as an Explanatory Mechanism for Poor Outcomes in Treatment for Substance Use Disorders. Lincoln: University of Nebraska (2015).

[32] Kelly EL Reed MK Schoenauer KM Smith K Scalia-Jackson K Kay Hill S. A Qualitative Exploration of the Functional, Social, and Emotional Impacts of the COVID-19 Pandemic on People Who Use Drugs. Int J Environ Res Public Health. (2022) 19:9751. doi: 10.3390/ijerph19159751 35955107 PMC9367729

[33] Conway FN Samora J Brinkley K Jeong H Clinton N Claborn KR. Impact of COVID-19 among people who use drugs: A qualitative study with harm reduction workers and people who use drugs. Harm Reduct J. (2022) 19:72. doi: 10.1186/s12954-022-00653-1 35780109 PMC9250267

[34] Moore DCBC Nehab MF Camacho KG Reis AT Junqueira-Marinho MDF Abramov DM. Low COVID-19 vaccine hesitancy in Brazil. Vaccine. (2021) 39:6262–8. doi: 10.1016/j.vaccine.2021.09.013 PMC8421107 34535318

[35] Substance Abuse and Mental Health Services Administration (SAMHSA). 2022 National Survey on Drug Use and Health (NSDUH) Detailed Tables. Rockville (MD: U.S. Department of Health and Human Services (2022). Available at: https://www.samhsa.gov/data/report/2022-nsduh-detailed-tables. Report No.: 2022 National Release (Accessed August 08, 2024).

[36] Humeniuk R Ali R Babor T Souza-Formigoni MLO de Lacerda RB Ling W. A randomized controlled trial of a brief intervention for illicit drugs linked to the Alcohol, Smoking and Substance Involvement Screening Test (ASSIST) in clients recruited from primary health-care settings in four countries. Addiction. (2012) 107:957–66. doi: 10.1111/j.1360-0443.2011.03740.x 22126102

[37] Brasil, Ministério da Saúde, Secretaria de Vigilância em Saúde e Ambiente, Departamento de Análise Epidemiológica e Vigilância de Doenças Não Transmissíveis. Vigitel Brazil 2023: surveillance of risk and protective factors for chronic diseases by telephone survey: estimates of frequency and sociodemographic distribution of risk and protective factors for chronic diseases in the capitals of the 26 Brazilian states and the Federal District in 2023. 1st ed. Brasília, DF: Ministério da Saúde (2023). 133 p. Available at: https://www.gov.br/saude/pt-br/centrais-de-conteudo/publicacoes/svsa/vigitel/vigitel-brasil-2023-vigilancia-de-fatores-de-risco-e-protecao-para-doencas-cronicas-por-inquerito-telefonico (Accessed April 17, 2024).

[38] Brasil, Ministério da Saúde, Secretaria de Vigilância em Saúde e Ambiente, Departamento de Análise Epidemiológica e Vigilância de Doenças Não Transmissíveis. Vigitel Brazil 2021: surveillance of risk and protective factors for chronic diseases by telephone survey: estimates of frequency and sociodemographic distribution of risk and protective factors for chronic diseases in the capitals of the 26 Brazilian states and the Federal District in 2021. 1st ed. Brasília, DF: Ministério da Saúde (2022). 131 p. Available at: https://www.gov.br/saude/pt-br/centrais-de-conteudo/publicacoes/svsa/vigitel/vigitel-brasil-2021-estimativas-sobre-frequencia-e-distribuicao-sociodemografica-de-fatores-de-risco-e-protecao-para-doencas-cronicas (Accessed April 17, 2024).

[39] Abe M Arima H Satoh A Okuda N Taniguchi H Nishi N. Marital status, household size, and lifestyle changes during the first COVID-19 pandemic: NIPPON DATA2010. PloS One. (2023) 18:e0283430. doi: 10.1371/journal.pone.0283430 36972241 PMC10042380

[40] Mitra S Bouck Z Larney S Zolopa C Høj S Minoyan N. The impact of the COVID-19 pandemic on people who use drugs in three Canadian cities: a cross-sectional analysis. Harm Reduct J. (2024) 21:94. doi: 10.1186/s12954-024-00996-x 38750575 PMC11097551

[41] Baum MK Tamargo JA Diaz-Martinez J Delgado-Enciso I Meade CS Kirk GD. HIV, psychological resilience, and substance misuse during the COVID-19 pandemic: A multi-cohort study. Drug Alcohol Depend. (2022) 231:109230. doi: 10.1016/j.drugalcdep.2021.109230 34998257 PMC8704725

[42] Calegaro VC Ramos-Lima LF Hoffmann MS Zoratto G Kerber N Costa FCD. Closed doors: Predictors of stress, anxiety, depression, and PTSD during the onset of COVID-19 pandemic in Brazil. J Affect Disord. (2022) 310:441–51. doi: 10.1016/j.jad.2022.05.052 PMC9107931 35569607

[43] Salari N Hosseinian-Far A Jalali R Vaisi-Raygani A Rasoulpoor S Mohammadi M. Prevalence of stress, anxiety, depression among the general population during the COVID-19 pandemic: a systematic review and meta-analysis. Glob Health. (2020) 16:57. doi: 10.1186/s12992-020-00589-w PMC7338126 32631403

[44] Christiansen DM McCarthy MM Seeman MV. Where Sex Meets Gender: How Sex and Gender Come Together to Cause Sex Differences in Mental Illness. Front Psychiatry. (2022) 13:856436. doi: 10.3389/fpsyt.2022.856436 35836659 PMC9273892

[45] Fountoulakis KN Vrublevska J Abraham S Adorjan K Ahmed HU Alarcón RD. Non-binary gender, vulnerable populations and mental health during the COVID-19 pandemic: Data from the COVID-19 MEntal health inTernational for the general population (COMET-G) study. J Affect Disord. (2024) 352:536–51. doi: 10.1016/j.jad.2024.02.050 38382816

[46] Manchia M Gathier AW Yapici-Eser H Schmidt MV De Quervain D Van Amelsvoort T. The impact of the prolonged COVID-19 pandemic on stress resilience and mental health: A critical review across waves. Eur Neuropsychopharmacol. (2022) 55:22–83. doi: 10.1016/j.euroneuro.2021.10.864 34818601 PMC8554139

[47] Apóstolo JLA Figueiredo MH Mendes AC Rodrigues MA. Depression, anxiety and stress in primary health care users. Rev Lat Am Enfermagem. (2011) 19:348–53. doi: 10.1590/S0104-11692011000200017 21584382

[48] BRASIL. LEI N° 13.840, DE 5 DE JUNHO DE 2019 - Nova Lei de Drogas. In: Civil Code, 13840. Brasília, DF. (2019). Available at: https://www.planalto.gov.br/ccivil_03/_ato2019-2022/2019/lei/l13840.htm (Accessed October 19, 2024).

[49] Salomone A Vincenti M. Detecting novel psychoactive substances around the world. Curr Opin Psychiatry. (2024) 37:258–63. doi: 10.1097/YCO.0000000000000939 38818825

[50] Wagmann L Maurer HH. Bioanalytical Methods for New Psychoactive Substances. In: Maurer HH Brandt SD, editors. New Psychoactive Substances : Pharmacology, Clinical, Forensic and Analytical Toxicology. Springer International Publishing, Cham (2018). p. 413–39. doi: 10.1007/164_2017_83 29374833

[51] Rinaldi R Bersani G Marinelli E Zaami S. The rise of new psychoactive substances and psychiatric implications: A wide-ranging, multifaceted challenge that needs far-reaching common legislative strategies. Hum Psychopharmacol. (2020) 35:e2727. doi: 10.1002/hup.v35.3 32144953

[52] Lo Faro AF Berardinelli D Cassano T Dendramis G Montanari E Montana A. New Psychoactive Substances Intoxications and Fatalities during the COVID-19 Epidemic. Biology. (2023) 12:273. doi: 10.3390/biology12020273 36829550 PMC9953068

[53] Font-Mayolas S Calvo F. Polydrug Definition and Assessment: The State of the Art. Int J Environ Res Public Health. (2022) 19:13542. doi: 10.3390/ijerph192013542 36294127 PMC9602920

[54] Wagner KD Fiuty P Page K Tracy EC Nocera M Miller CW. Prevalence of fentanyl in methamphetamine and cocaine samples collected by community-based drug checking services. Drug Alcohol Depend. (2023) 252:110985. doi: 10.1016/j.drugalcdep.2023.110985 37826988 PMC10688611

[55] UNODC. World Drug Report 2022. Vienna: United Nations (2022).

[56] Karch SB Busardò FP Vaiano F Portelli F Zaami S Bertol E. Levamisole adulterated cocaine and pulmonary vasculitis: Presentation of two lethal cases and brief literature review. Forensic Sci Int. (2016) 265:96–102. doi: 10.1016/j.forsciint.2016.01.015 26855022

[57] Wang L Jeong EM Krebs E Evans E Huang D Liu L. Polydrug use and its association with drug treatment outcomes among primary heroin, methamphetamine, and cocaine users. Int J Drug Policy. (2017) 49:32–40. doi: 10.1016/j.drugpo.2017.07.009 28888099 PMC5681890

[58] Substance Abuse and Mental Health Services Administration (US) Office of the Surgeon General (US). Prevention Programs and Policies. In: Facing Addiction in America: The Surgeon General’s Report on Alcohol, Drugs, and Health. Rockville, Maryland: US Department of Health and Human Services (2016). Available at: https://www.ncbi.nlm.nih.gov/books/NBK424850/. 28252892

[59] Office of National Drug Control Policy. National Drug Control Strategy. Washington, DC: The White House (2024). Available at: https://www.whitehouse.gov/wp-content/uploads/2024/05/2024-National-Drug-Control-Strategy.pdf (Accessed December 23, 2024).

[60] Simha S Ahmed Y Brummett CM Waljee JF Englesbe MJ Bicket MC. Impact of the COVID-19 pandemic on opioid overdose and other adverse events in the USA and Canada: a systematic review. Reg Anesth Pain Med. (2023) 48:37–43. doi: 10.1136/rapm-2022-103591 36202619

[61] Ramirez-Santana M. Limitations and Biases in Cohort Studies. In: Cohort Studies in Health Sciences. London, UK: IntechOpen (2018). Available at: https://www.intechopen.com/chapters/59393 (Accessed August 28, 2024).

