# Supplementary material for: A cohort-based assessment of drug use trends during the COVID-19 pandemic: relationship with mood and sociodemographic factors in Brazil
Source: Front Psychiatry. 2025 Jan 30;16:1514365. doi: 10.3389/fpsyt.2025.1514365 (PMC11841454; doi:10.3389/fpsyt.2025.1514365)
Supplement: Supplementary file 1 [file Table1.docx]

Supplementary Material

# Supplementary Data

Table S1. ASSIST and DASS-21 scores’ Spearman’s Correlation Coefficients and statistical significance, for all three inquiry phases (C1, C2 and C3).

| **Spearman's Correlation Coefficient** | | **C1** | | | **C2** | | **C3** | | |
| --- | --- | --- | --- | --- | --- | --- | --- | --- | --- |
|  |  | r | *P* | r | | *P* | | r | *P* |
| **ALCOHOL** | **Depression** | 0,278 | 0,000 | 0,254 | | 0,000 | | 0,231 | 0,000 |
|  | **Anxiety** | 0,258 | 0,000 | 0,276 | | 0,000 | | 0,266 | 0,000 |
|  | **Stress** | 0,273 | 0,000 | 0,245 | | 0,000 | | 0,251 | 0,000 |
| **TOBACCO** | **Depression** | 0,201 | 0,000 | 0,201 | | 0,000 | | 0,232 | 0,000 |
|  | **Anxiety** | 0,255 | 0,000 | 0,267 | | 0,000 | | 0,286 | 0,000 |
|  | **Stress** | 0,209 | 0,000 | 0,203 | | 0,000 | | 0,215 | 0,000 |
| **CANNABIS** | **Depression** | 0,175 | 0,000 | 0,131 | | 0,001 | | 0,186 | 0,000 |
|  | **Anxiety** | 0,194 | 0,000 | 0,214 | | 0,000 | | 0,232 | 0,000 |
|  | **Stress** | 0,145 | 0,000 | 0,118 | | 0,002 | | 0,158 | 0,000 |
| **COCAINE/ CRACK** | **Depression** | 0,154 | 0,001 | 0,147 | | 0,031 | | 0,220 | 0,002 |
|  | **Anxiety** | 0,165 | 0,001 | 0,161 | | 0,018 | | 0,260 | 0,000 |
|  | **Stress** | 0,129 | 0,008 | 0,107 | | 0,116 | | 0,224 | 0,001 |
| **AMPHETAM./ ECSTASY** | **Depression** | 0,177 | 0,000 | 0,329 | | 0,000 | | 0,189 | 0,001 |
|  | **Anxiety** | 0,141 | 0,001 | 0,342 | | 0,000 | | 0,181 | 0,002 |
|  | **Stress** | 0,121 | 0,004 | 0,341 | | 0,000 | | 0,177 | 0,002 |
| **HYPNOTICS** | **Depression** | 0,290 | 0,000 | 0,296 | | 0,000 | | 0,366 | 0,000 |
|  | **Anxiety** | 0,355 | 0,000 | 0,441 | | 0,000 | | 0,312 | 0,000 |
|  | **Stress** | 0,345 | 0,000 | 0,325 | | 0,000 | | 0,317 | 0,000 |
| **HALLUCINOGENS** | **Depression** | 0,135 | 0,000 | 0,186 | | 0,001 | | 0,157 | 0,008 |
|  | **Anxiety** | 0,115 | 0,000 | 0,170 | | 0,004 | | 0,127 | 0,031 |
|  | **Stress** | 0,127 | 0,000 | 0,201 | | 0,001 | | 0,129 | 0,029 |
| **OPIOIDS** | **Depression** | 0,431 | 0,000 | 0,266 | | 0,023 | | 0,255 | 0,030 |
|  | **Anxiety** | 0,358 | 0,000 | 0,374 | | 0,001 | | 0,299 | 0,010 |
|  | **Stress** | 0,353 | 0,000 | 0,270 | | 0,021 | | 0,243 | 0,037 |

Coefficient interpretations were considered as follows: discrete: 0-0.20; regular: 0.20-0.60; moderate: 0.60-0.80; near perfect: 0.80-1.00. Coefficients and P-values were colored in different scales of colors to better represent strength and significance. The coefficients were painted from a scale of light yellow (discrete correlation), pale turquoise shades (regular correlation), and turquoise (above 0.40). Significance was colored in a scale of light yellow (> 0.05); light green tones (0.03 and lower); and soft green (⟞0.00).

Table S2. Prevalence of drug users by sociodemographic factors and time points (C1, C2 and C3), for the most relevant drugs in Brazil.

| **Sociodemo- graphics** | **Time** | **ALCOHOL** | **%** | **TOBACCO** | **%** | **CANNABIS** | **%** | **HYPNOTICS** | **%** | **COCAINE** | **%** |
| --- | --- | --- | --- | --- | --- | --- | --- | --- | --- | --- | --- |
|  | **C1** | 201 | 93.9 | 105 | 49.1 | 115 | 53.7 | 28 | 13.1 | 35 | 16.4 |
| **Male** | **C2** | 182 | 85.0 | 94 | 43.9 | 89 | 41.6 | 25 | 11.7 | 36 | 16.8 |
|  | **C3** | 190 | 88.8 | 107 | 50.0 | 102 | 47.7 | 25 | 11.7 | 31 | 14.5 |
| **Female** |  |  |  |  |  |  |  |  |  |  |  |
|  | **C1** | 478 | 89.5 | 185 | 34.6 | 170 | 31.8 | 90 | 16.9 | 50 | 9.4 |
|  | **C2** | 459 | 86.0 | 180 | 33.7 | 159 | 29.8 | 84 | 15.7 | 44 | 8.2 |
|  | **C3** | 457 | 85.6 | 188 | 35.2 | 188 | 35.2 | 89 | 16.7 | 53 | 9.9 |
| **Single/ Divorced/ Widowed** |  | **ALCOHOL** | **%** | **TOBACCO** | **%** | **CANNABIS** | **%** | **HYPNOTICS** | **%** | **COCAINE** | **%** |
|  | **C1** | 455 | 90.3 | 213 | 42.3 | 212 | 42.1 | 83 | 16.5 | 56 | 11.1 |
|  | **C2** | 426 | 84.5 | 204 | 40.5 | 189 | 37.5 | 75 | 14.9 | 60 | 11.9 |
|  | **C3** | 436 | 86.5 | 225 | 44.6 | 214 | 42.5 | 83 | 16.5 | 60 | 11.9 |
| **Married/ Stable Union** |  |  |  |  |  |  |  |  |  |  |  |
|  | **C1** | 229 | 91.2 | 82 | 32.7 | 79 | 31.5 | 38 | 15.1 | 32 | 12.7 |
|  | **C2** | 220 | 87.6 | 75 | 29.9 | 65 | 25.9 | 37 | 14.7 | 23 | 9.2 |
|  | **C3** | 216 | 86.1 | 76 | 30.3 | 82 | 32.7 | 34 | 13.5 | 28 | 11.2 |
| **Incomplete Secondary Education** |  | **ALCOHOL** | **%** | **TOBACCO** | **%** | **CANNABIS** | **%** | **HYPNOTICS** | **%** | **COCAINE** | **%** |
|  | **C1** | 3 | 100 | 0 | 0.0 | 0 | 0.0 | 1 | 33.3 | 0 | 0.0 |
|  | **C2** | 2 | 66.7 | 0 | 0.0 | 0 | 0.0 | 0 | 0.0 | 0 | 0.0 |
|  | **C3** | 3 | 100 | 1 | 33.3 | 0 | 0.0 | 0 | 0.0 | 0 | 0.0 |
| **Secondary Education** |  |  |  |  |  |  |  |  |  |  |  |
|  | **C1** | 180 | 92.3 | 111 | 56.9 | 106 | 54.4 | 47 | 24.1 | 38 | 19.5 |
|  | **C2** | 163 | 83.6 | 109 | 55.9 | 96 | 49.2 | 39 | 20.0 | 37 | 19.0 |
|  | **C3** | 173 | 88.7 | 120 | 61.5 | 115 | 59.0 | 48 | 24.6 | 38 | 19.5 |
| **Higher Education** |  |  |  |  |  |  |  |  |  |  |  |
|  | **C1** | 502 | 90.1 | 184 | 33.0 | 185 | 33.2 | 73 | 13.1 | 50 | 9.0 |
|  | **C2** | 482 | 86.5 | 170 | 30.5 | 158 | 28.4 | 73 | 13.1 | 46 | 8.3 |
|  | **C3** | 477 | 85.6 | 180 | 32.3 | 181 | 32.5 | 69 | 12.4 | 50 | 9.0 |
| **Up to R$ 750,00** |  | **ALCOHOL** | **%** | **TOBACCO** | **%** | **CANNABIS** | **%** | **HYPNOTICS** | **%** | **COCAINE** | **%** |
|  | **C1** | 39 | 92.9 | 24 | 57.1 | 22 | 52.4 | 10 | 23.8 | 9 | 21.4 |
|  | **C2** | 38 | 90.5 | 22 | 52.4 | 21 | 50.0 | 10 | 23.8 | 11 | 26.2 |
|  | **C3** | 40 | 95.2 | 26 | 61.9 | 25 | 59.5 | 10 | 23.8 | 11 | 26.2 |
| **From R$ 751,00 to 1.500,00** |  |  |  |  |  |  |  |  |  |  |  |
|  | **C1** | 57 | 93.4 | 32 | 52.5 | 36 | 59.0 | 19 | 31.1 | 15 | 24.6 |
|  | **C2** | 55 | 90.2 | 33 | 54.1 | 31 | 50.8 | 17 | 27.9 | 14 | 23.0 |
|  | **C3** | 54 | 88.5 | 33 | 54.1 | 38 | 62.3 | 14 | 23.0 | 18 | 29.5 |
| **From R$ 1.501,00 to R$ 3.000,00** |  |  |  |  |  |  |  |  |  |  |  |
|  | **C1** | 140 | 94.0 | 73 | 49.0 | 81 | 54.4 | 26 | 17.4 | 21 | 14.1 |
|  | **C2** | 133 | 89.3 | 71 | 47.7 | 77 | 51.7 | 24 | 16.1 | 24 | 16.1 |
|  | **C3** | 138 | 92.6 | 84 | 56.4 | 88 | 59.1 | 27 | 18.1 | 25 | 16.8 |
| **From R$ 3.001,00 to R$ 6.000,00** |  |  |  |  |  |  |  |  |  |  |  |
|  | **C1** | 180 | 89.6 | 71 | 35.3 | 70 | 34.8 | 30 | 14.9 | 22 | 10.9 |
|  | **C2** | 176 | 87.6 | 74 | 36.8 | 74 | 36.8 | 28 | 13.9 | 14 | 7.0 |
|  | **C3** | 178 | 88.6 | 80 | 39.8 | 79 | 39.3 | 30 | 14.9 | 16 | 8.0 |
| **From R$ 6.001,00 to R$ 9.000,00** |  |  |  |  |  |  |  |  |  |  |  |
|  | **C1** | 97 | 89.8 | 39 | 36.1 | 34 | 31.5 | 14 | 13.0 | 8 | 7.4 |
|  | **C2** | 96 | 88.9 | 36 | 33.3 | 26 | 24.1 | 9 | 8.3 | 11 | 10.2 |
|  | **C3** | 98 | 90.7 | 37 | 34.3 | 30 | 27.8 | 9 | 8.3 | 10 | 9.3 |
| **More than R$9.000,00** |  |  |  |  |  |  |  |  |  |  |  |
|  | **C1** | 172 | 88.7 | 56 | 28.9 | 48 | 24.7 | 23 | 11.9 | 13 | 6.7 |
|  | **C2** | 173 | 89.2 | 55 | 28.4 | 41 | 21.1 | 27 | 13.9 | 13 | 6.7 |
|  | **C3** | 168 | 86.6 | 58 | 29.9 | 54 | 27.8 | 29 | 14.9 | 15 | 7.7 |
| **Low Social Distancing** |  | **ALCOHOL** | **%** | **TOBACCO** | **%** | **CANNABIS** | **%** | **HYPNOTICS** | **%** | **COCAINE** | **%** |
|  | **C1** | 28 | 80.0 | 15 | 42.9 | 18 | 51.4 | 6 | 17.1 | 4 | 11.4 |
|  | **C2** | 29 | 82.9 | 16 | 45.7 | 17 | 48.6 | 6 | 17.1 | 2 | 5.7 |
|  | **C3** | 30 | 85.7 | 18 | 51.4 | 19 | 54.3 | 7 | 20.0 | 6 | 17.1 |
| **Medium Social Distancing** |  |  |  |  |  |  |  |  |  |  |  |
|  | **C1** | 267 | 92.4 | 114 | 39.4 | 121 | 41.9 | 53 | 18.3 | 40 | 13.8 |
|  | **C2** | 261 | 90.3 | 115 | 39.8 | 118 | 40.8 | 50 | 17.3 | 40 | 13.8 |
|  | **C3** | 261 | 90.3 | 123 | 42.6 | 130 | 45.0 | 57 | 19.7 | 38 | 13.1 |
| **High Social Distancing** |  |  |  |  |  |  |  |  |  |  |  |
|  | C1 | 390 | 90.5 | 166 | 38.5 | 152 | 35.3 | 62 | 14.4 | 44 | 10.2 |
|  | C2 | 381 | 88.4 | 160 | 37.1 | 135 | 31.3 | 59 | 13.7 | 45 | 10.4 |
|  | C3 | 385 | 89.3 | 177 | 41.1 | 165 | 38.3 | 55 | 12.8 | 51 | 11.8 |

An analysis to control the effect of covariables was performed for each drug class. The ones which showed an interaction between covariable and time were alcohol (Income), amphetamines/ecstasy (Gender and Education) and hypnotics (Education and Income). The effect of covariables and their pairwise method comparisons will be described hereafter. The absolute number of females is higher than males, higher than 60% for most drugs, although cocaine showed less of a gap between sexes in the first time point, and changes towards the major pattern afterwards. Rather not answer presented very little sample sizes, and did not reach statistical power for analysis. The marital status showed similar frequencies for all drugs, and being Single/Divorced/Widowed obtained higher prevalence among them, in all cohorts. Marital status did not show interaction with Time for any drug class. The drugs which presented effect of the covariable and/or time were alcohol (covariable: *P* = 0.005; time: *P* = 0.000), tobacco (covariable: *P* = 0.001), cannabis (covariable: *P* = 0.008; time: *P* = 0.020), cocaine (covariable: *P* = 0.020), hallucinogens (covariables: *P* = 0.004; time: *P* = 0.000). People who did not live in union, meaning Single/Divorced/Widowed, reported greater scores for the aforementioned drugs than people who lived in union, or Married/Stable union.
